# Does maladaptive microvascular flow redistribution cause tissue hypoxia in fibromyalgia?

**DOI:** 10.1097/PR9.0000000000001459

**Published:** 2026-08-14

**Authors:** Uri Gabbay, Or Carmi

**Affiliations:** a Gray Faculty of Medicine, Tel Aviv University, Tel Aviv, Israel; b Quality Unit, Rabin Medical Center, Petah Tikva, Israel; c Meir Medical Center, Kfar-Saba, Israel

**Keywords:** Fibromyalgia, Maladaptive Capillary Shunting, Metarteriole Thoroughfare Channel, Microcirculatory Dysfunction, Nociceptor Sensitization, Tissue hypoxia

## Abstract

Fibromyalgia is a chronic pain syndrome characterized by widespread pain, fatigue, and cognitive complaints often described by patients as “brain fog”. In the absence of specific physiological, laboratory, or imaging markers, diagnosis remains clinical. Despite extensive investigation, its underlying mechanisms remain unclear. While altered pain processing (known also as “central sensitization”) has long dominated explanatory models, accumulating evidence suggests contributory peripheral physiological abnormalities. Reported findings include normal arterial oxygenation alongside reduced tissue oxygen saturation, relatively elevated venous oxygen saturation, and increased tissue lactate, collectively indicating impaired tissue oxygen extraction. Studies examining microcirculatory abnormalities as a causal mechanism have yielded inconsistent results. We propose that fibromyalgia may involve maladaptive capillary-level blood flow distribution prioritizing metarteriolar thoroughfare channels, physiological low-resistance microvascular pathways involved in vasomotion and flow regulation. If this regulatory mechanism becomes maladaptive, blood flow may preferentially bypass exchange capillaries, reducing local oxygen availability below metabolic demand and producing intermittent tissue hypoxia. This hypoxic stress may contribute directly to pain, tenderness, and fatigue and may arise locally or through impaired autonomic regulation of vasomotion. This model offers a potential explanation for the paradox of tissue hypoxia despite preserved systemic oxygen delivery and generates testable physiological predictions. However, current evidence remains circumstantial, and the proposed mechanism requires empirical validation.

## Abstract 2

The authors propose that maladaptive functional microvascular flow redistribution toward non-nutritive pathways, at the expense of exchange capillary perfusion, impairs tissue oxygenation and induces localized hypoxic stress, which may contribute to pain and fatigue in fibromyalgia.

## 1. Introduction

Fibromyalgia (FM) affects approximately 2% to 4% of the population and is more common in women.^5,14^ It is characterized by chronic widespread pain, fatigue, and cognitive symptoms typically described as “brain fog.”^5,14,31^ Diagnosis remains clinical, based on symptom patterns and exclusion of alternative explanations, consistent with the 2016 American College of Rheumatology criteria.^33^ Despite decades of investigation, the pathophysiology of FM remains incompletely understood. Altered oxygen saturation patterns have been described in several studies.

Current explanatory models emphasize altered pain processing, involving abnormal central nervous system activity that amplifies pain perception.^26,31^ Numerous peripheral and central mechanisms have been proposed, including autonomic dysfunction, mitochondrial abnormalities, immune signaling alterations, and neuroendocrine dysregulation (Table 1). While each of these mechanisms may contribute to impaired oxygen utilization, none fully account for reports of preserved systemic oxygen delivery with impaired tissue extraction. This gap supports consideration of microvascular flow redistribution as a complementary mechanism.

**Table 1:** Suggested mechanisms for fibromyalgia by physiological anatomy, namely central and peripheral mechanisms, in which we cited these references.

Mechanism type	Specific mechanism	References
Central mechanisms	Pain modulation dysfunction: dysregulated descending inhibitory control and enhanced pain facilitation pathways	5, 14, 31, 32
	Altered pain processing: amplified neural signaling within the CNS leading to pain hypersensitivity	5, 14, 26, 31–33
	Neurophysiological dysregulation: altered cortical excitability and pain network connectivity	26, 31–33
	Neuroinflammation: microglial and astrocyte activation in the CNS	2
	Neurotransmitter dysregulation: altered serotonin, dopamine, and norepinephrine signaling	5, 14, 26, 31
	Autonomic dysfunction (dysautonomia): abnormal integration of autonomic and sensory signaling (particularly sympathetic overactivity)	9, 16
	Altered stress response (HPA-axis dysregulation): abnormal cortisol and stress responsiveness	14, 26
Peripheral mechanisms	Small fiber abnormality: structural (reduced intraepidermal fiber density) or functional (impaired sensory fiber function contributing to pain and dysesthesia)	13, 24
	Vascular dysregulation: impaired endothelial function or vasomotion	3, 12, 21, 35
	Localized tissue hypoxia: reduced oxygen delivery due to maldistribution of capillary flow	8, 19, 29, 30
	Metabolic disturbances: altered muscle metabolism, elevated lactate, or impaired energy utilization	10, 20
	Tissue oxygenation abnormalities: imbalance between oxygen delivery and utilization	8, 19, 29, 30
	Mitochondrial dysfunction: impaired ATP production and oxidative stress	1, 27
	Nociceptor sensitization: increased peripheral nerve excitability due to hypoxia or inflammation	3, 35
	Immune dysregulation: abnormal cytokine profiles and low-grade inflammation or immune-mediated microvascular changes	6, 7, 18, 23
	Neuromuscular impairment: altered muscle activation and endurance	10
	Fascial abnormalities: altered fascial perfusion and stiffness affecting nociceptive input	4, 7, 25

Reference 27 (Pirri et al.) pertains to Complex Regional Pain Syndrome (CRPS) rather than fibromyalgia, cited here for comparison of fascial or microvascular mechanisms.

Attempts to identify objective biomarkers in fibromyalgia have yielded heterogeneous results. Evidence includes mitochondrial abnormalities correlated with symptom severity and proteomic and genetic associations with oxidative stress, inflammation, and metabolic regulation.^9,12,16,21,35^ However, these findings do not fully explain impaired oxygen utilization. This article presents a hypothesis-driven conceptual synthesis.

## 2. Altered oxygen utilization in fibromyalgia

Reported oxygenation patterns include reduced tissue oxygen saturation, relatively elevated peripheral venous oxygen saturation, and increased tissue lactate despite preserved systemic oxygen delivery.^3,8,29,30^

Elvin et al.^8^ demonstrated reduced muscle perfusion during exercise, suggesting localized ischemia and metabolic stress. Shang et al.^30^ reported impaired oxygen extraction and delayed postexercise reoxygenation. Rubio-Zarapuz et al.^29^ observed reduced muscle oxygen saturation at rest and during exertion, implicating microcirculatory dysfunction and autonomic dysregulation. Aloisi et al.^3^ highlighted impaired vascular regulation as a potential contributor to reduced tissue oxygen availability.

This pattern presents a physiological paradox: biochemical and functional features of localized hypoxia occur despite preserved systemic oxygen delivery. Collectively, these observations suggest impaired oxygen extraction at the microcirculatory level, although a single causal mechanism has not been identified.

## 3. Potential mechanisms of impaired oxygen extraction

Several mechanisms could theoretically contribute to impaired tissue oxygen utilization in FM. Potential contributors include hypoperfusion, capillary rarefaction, and endothelial dysfunction. Mitochondrial dysfunction may impair intracellular oxygen utilization but does not directly explain impaired oxygen extraction.

However, mitochondrial inefficiency alone cannot readily explain impaired oxygen exchange at the capillary–tissue interface. An upstream disturbance in microvascular regulation may therefore be involved.

One potential mechanism is maladaptive redistribution of microvascular flow rather than structural abnormality, in which blood preferentially flows through low-resistance pathways that bypass exchange capillaries. This would limit effective oxygen exchange despite preserved arterial inflow. Such mechanisms are not mutually exclusive and may coexist across patient subsets.

## 4. Evidence of microcirculatory abnormalities

Several studies support microcirculatory involvement in FM, although findings remain heterogeneous. Reduced capillary density has been reported in subsets of patients.^19^ Altered fascial perfusion and nutrient delivery have also been described.^10,20,27^ Albrecht et al.^1^ reported excessive sensory innervation surrounding arterioles and capillaries, suggesting abnormal neurovascular regulation.

Nailfold capillaroscopy studies demonstrate reduced density, abnormal morphology, and impaired flow dynamics, findings consistent with impaired vascular regulation and intermittent hypoxia.^23^ Dysregulated sympathetic control of microcirculation has also been proposed as a contributor to exertional pain and fatigue.^6,18^

Collectively, these findings suggest that abnormalities of microvascular regulation may contribute to impaired tissue oxygen utilization in FM.

## 5. Normal microcirculatory architecture and regulation

The capillary network includes exchange capillaries and upstream flow-regulating structures that dynamically regulate blood flow distribution through vasomotion, intermittently perfusing exchange capillaries.

Blood flow may be redistributed between nutritive and non-nutritive pathways through arteriolar control mechanisms in skeletal muscle,^34^ and through metarteriolar thoroughfare channels in skin and connective tissues. This redistribution may enable functional shunting without fixed anatomical shunts and is modulated by vasoconstrictors and sympathetic activity. Local metabolic signals (eg, hypoxia and metabolic byproducts) promote capillary recruitment. Under normal conditions, vasomotor and metabolic signals balance exchange perfusion and shunt flow.^7^

## 6. The proposed hypothesis

We propose that a normally adaptive capillary flow distribution mechanism becomes maladaptive in fibromyalgia. Preferential flow bypassing exchange capillary recruitment could limit tissue oxygen availability despite normal arterial oxygenation.

This redistribution could produce mild or intermittent localized hypoxia that is insufficient to cause ischemic injury but sufficient to reduce tissue oxygen saturation, elevate tissue lactate, and increase collecting venous oxygen saturation.

The proposed model integrates impaired oxygen extraction, microvascular abnormalities, dysautonomia, and metabolic alterations.

**Figure 1.**
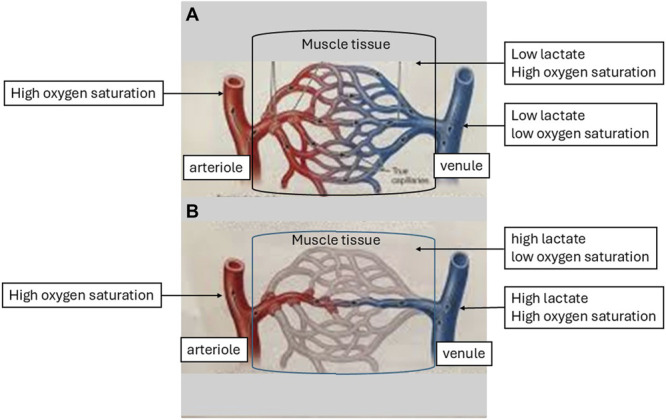
Conceptual schematic model. A. Under physiological conditions, upstream regulatory structures dynamically distribute blood between exchange capillaries and non-nutritive channels to match local metabolic demand, ensuring adequate tissue oxygenation and preventing lactate accumulation. B. Proposed mechanism in fibromyalgia: impaired upstream regulation → redistribution of flow toward non-nutritive pathways → reduced capillary perfusion → intermittent tissue hypoxia → nociceptor sensitization and generation of pain and tenderness. This schematic is conceptual and illustrative rather than anatomically precise.

Figure 1 illustrates the proposed redistribution mechanism, including exchange capillaries and upstream arteriolar regulation under normal vs maladaptive flow distribution.

Recurrent peripheral hypoxic stress may activate and sensitize nociceptors through mediators such as inflammatory and metabolic signals. These mechanisms could contribute to pain, tenderness, and fatigue.

The initiating process may involve either peripheral or central mechanisms. Peripheral microvascular dysregulation may generate afferent nociceptive signals, whereas central maladaptive processing may alter sympathetic outflow and disrupt vasomotion regulation. Clinically, this may manifest as exertional intolerance, activity-related pain, and delayed recovery.

Importantly, the hypothesis does not require permanent structural shunting. Instead, transient dysregulation could intermittently redistribute microvascular flow toward non-nutritive pathways.

This model is not intended to replace existing central or peripheral mechanisms but to complement them by addressing a specific physiological paradox related to oxygen utilization.

## 7. Supporting evidence

Evidence of impaired muscle oxygen utilization and metabolic dysfunction in FM has been reported using magnetic resonance spectroscopy, electron microscopy, and exercise testing.^4,16,25^ Dysautonomia, frequently observed in FM, may influence vascular tone and exacerbate abnormal microvascular flow distribution.^22,28^

Analogous oxygenation patterns have been described in myalgic encephalomyelitis/chronic fatigue syndrome and postural orthostatic tachycardia syndrome, where relatively elevated regional or peripheral venous oxygen saturation reflects impaired tissue oxygen extraction.^17^ Similar shunting mechanisms have been proposed in diabetic microvascular disease.^11^

Comparable patterns of impaired oxygen extraction despite preserved oxygen delivery have been reported in conditions such as sepsis. In sepsis, these abnormalities reflect systemic inflammation and endothelial injury. By contrast, the mechanism proposed for FM is localized and peripheral, potentially involving regional microcirculatory dysregulation without systemic inflammation.^15^

Small fiber pathology reported in subsets of patients with FM may further contribute to autonomic dysregulation of vascular tone and altered microvascular perfusion.

Although these findings support biological plausibility, they remain largely indirect and do not demonstrate capillary-level shunting directly. The proposed model should therefore be interpreted as an extrapolative physiological hypothesis derived from converging observations.

## 8. Testing the hypothesis

Testing requires integrated structural, neural, and endothelial assessments. Structural evaluation may include imaging techniques such as contrast-enhanced ultrasound or MRI.

Neural regulation may be assessed using autonomic measures such as heart rate variability.

Endothelial function may be evaluated using endothelial function testing (eg, flow-mediated dilation or near-infrared spectroscopy).

If correct, the model predicts reduced tissue oxygenation during demand, transient lactate elevation, and elevated venous oxygen saturation.

Therapies improving microcirculatory regulation would be expected to improve physiological measures and symptoms.

Testing limitations include invasive or indirect measurement methods, potential sampling inaccuracy, and small signal differences near imaging resolution limits. Microcirculatory abnormalities may also be localized or transient.

## 9. Implications

This hypothesis suggests that fibromyalgia may involve microcirculatory dysfunction in addition to altered central pain processing. Identifying abnormalities in tissue oxygen extraction could improve diagnostic confidence and explain the coexistence of preserved systemic oxygen delivery with features of hypoxia. The model also highlights potential therapeutic targets, including vascular regulation, angiogenesis, and autonomic modulation.

## 10. Limitations

The main limitation is reliance on indirect and heterogeneous evidence. Direct fibromyalgia-specific investigations examining capillary perfusion dynamics, venous oxygen patterns, and microvascular regulation are needed. Additional limitations include the inability to directly visualize capillary-level shunting, the heterogeneity of fibromyalgia, and the possibility that this mechanism may apply only to a subset of patients with fibromyalgia.

## 11. Conclusion

We propose that maladaptive redistribution of microvascular flow rather than structural abnormality may contribute to fibromyalgia by inducing recurrent localized hypoxic stress. This mechanism links impaired oxygen utilization with metabolic stress, nociceptor sensitization, autonomic dysregulation, and central pain amplification. The hypothesis generates testable predictions and may help reconcile diverse clinical and physiological observations in fibromyalgia.

## Disclosures

The authors have no conflict of interest to declare.
